# Meetings of Societies

**Published:** 1933

**Authors:** 


### Meetings of Societies

### Bristol Medico-Chirurgical Society

The fourth meeting of the session was held in the Physiological Lecture Theatre of the University, on Wednesday, 11th January, 1933, at 8.30 p.m., Mr. E. W. Hey Groves in the Chair.

X-rays were shown by Dr. F. J. Mayes and pathological specimens by Dr. A. L. Taylor.

Dr. A. T. Todd showed two cases of **pseudo-hypertrophic paralysis treated by sodium fluoride.** A discussion followed these cases.

Mr. Angell James and Dr. A. T. Todd read a paper on "**The Treatment of Asthma.**"

A discussion followed in which the President, Dr. Symes, Dr. Carleton, Dr. Perry, Dr. Gee, Mr. Wright, Dr. Birrell, Dr. Frank Bodman, Dr. Hector, and Professor Nixon spoke.

---

The fifth meeting of the session was held in the Physiological Lecture Theatre of the University, on Wednesday, 8th February, 1933, at 8.30 p.m., Mr. E. W. Hey Groves in the Chair.

Dr. A. D. Fraser and Dr. T. B. Wansbrough showed pathological specimens and X-rays.

The minutes of the previous meeting were read and confirmed.

The President introduced Mr. Rock Carling, who gave an address entitled "**Surgery for Pain.**"

Mr. C. Ferrier Walters proposed a vote of thanks to Mr. Rock Carling, which was seconded by Mr. Clifford Moore, and carried with acclamation.

Mr. Rock Carling replied.

